# Auto-Induction Effect of Chloroxoquinoline on the Cytochrome P450 Enzymes of Rats Associated with CYP 3A and 1A

**DOI:** 10.1371/journal.pone.0138875

**Published:** 2015-09-23

**Authors:** Xin Li, Ying Li, Wei Gong, Mei Yan Yang, Yang Yang, Zhi Ping Li, Yu Li Wang, Zhen Qing Zhang

**Affiliations:** 1 Beijing Institute of Pharmacology and Toxicology, Beijing, China; 2 Department of Pharmacy, Chinese PLA General Hospital, Beijing, China; Universidade Federal do Rio de Janeiro, BRAZIL

## Abstract

To investigate the auto-induction of cytochrome P450 (CYP450) by Chloroxoquinoline (CXL), a novel anticancer drug. Three experiments related to the induction of CYP450 were performed: a) *In vitro* use of the rat fresh hepatocytes model; b) *In vivo* ‘cocktail’ of CYP450 probe model; c) Pharmacokinetic (PK) study of the single and multiple doses. Some typical CYP enzyme probes and inducers were used in these experiments and were all determined by HPLC-MS/MS. The expression levels of CYP3A and CYP1A mRNA were analyzed by the real time polymerase chain reaction (RT-PCR) technique. The PK studies showed that the area under the curve (AUC_0-t_) and the peak concentration (C_max_) of the multiple doses were approximately 2.4-fold and 1.9-fold lower than those of the single dose, respectively (*p*< 0.05). Subsequent studies were conducted to study the possible induction of CXL on CYP 450. The *in vivo* ‘cocktail’ administration of CYP450 probe model indicated that 5 d pretreatment with CXL resulted in a mean 4.6 times increase in the metabolites/probe plasma ratios for CYP 3A and a 336% increase for CYP 1A than those of the negative control (*p*< 0.05). The induction effect of CXL on CYP450 was further evaluated on rat hepatocytes with four concentrations (1, 10, 50 and 100 μmol/L). Compared with the negative control, the mRNA levels of CYP 1A2 increased significantly in rat hepatocytes after treatment with 10, 50 and 100 μmol/L CXL (*p*< 0.05). While significant inductions of CYP 3A1 were observed in the entire treated groups. The results of the present study demonstrate enhanced and induced expression of CYP 3A and CYP 1A in response to CXL exposure in rats, suggesting that CXL is an auto-inducer of CYP 3A and CYP 1A.

## Introduction

**Fig 1:**
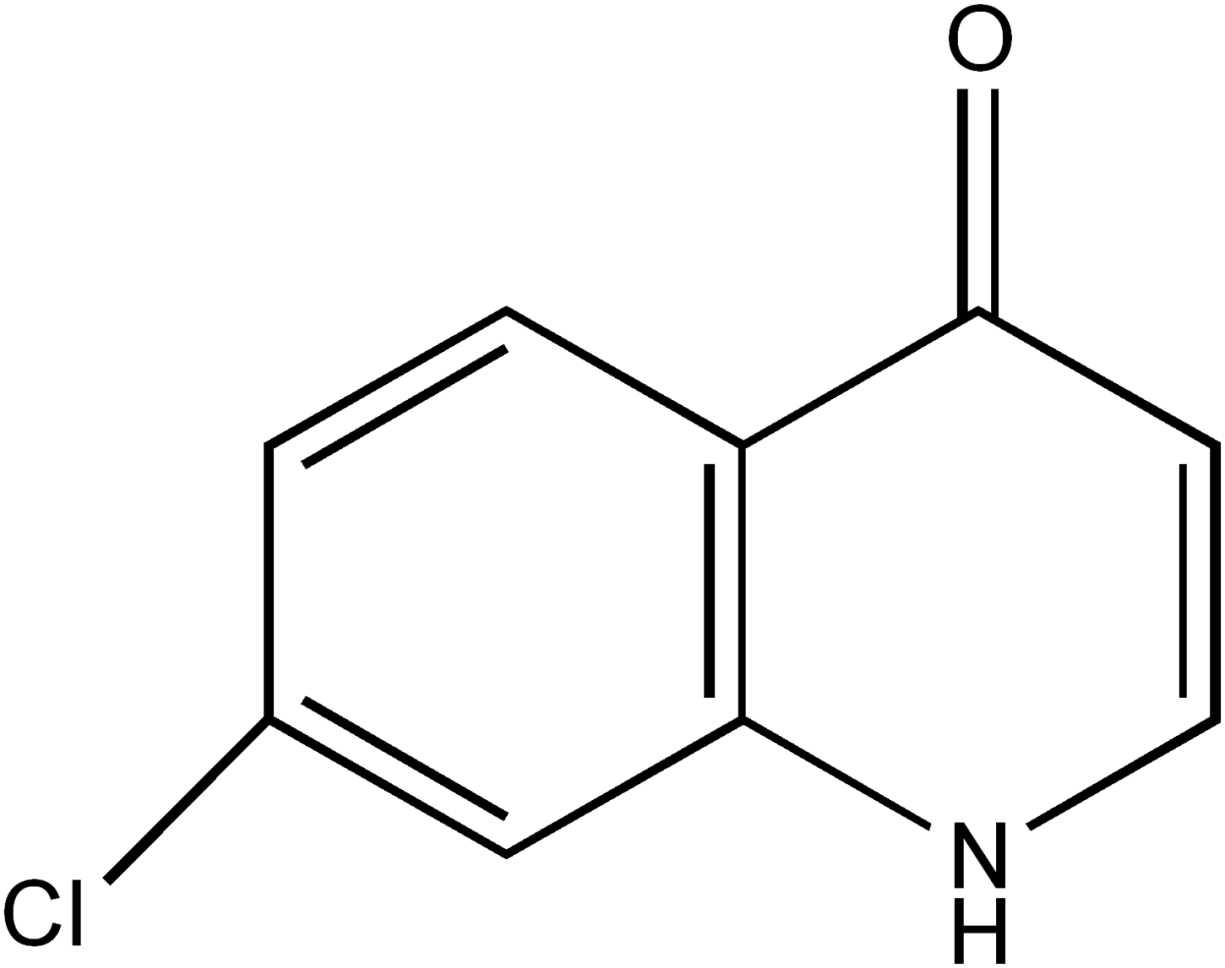
Chemical structure of chloroxoquinoline (7-chloro-4-keto-quinoline, CXL).

Chloroxoquinoline (CXL) [7-Chloro-4-keto-quinoline], is a novel anticancer drug, having a new chemical structure shown in Fig 1. In preclinical studies, CXL could enhance the radiation sensitivity of Lewis lung cancer cells and xenograft tumors in tumor-bearing mice [1]. In clinical studies, CXL exhibited better anticancer effects and lower toxicity compared to other commonly used anticancer drugs, such as a cyclophosphamide-cisplatin-adriamycin combined chemotherapy (CAP) regimen and an epirubicin-cisplatin-5-fluorouracil (ECF) regimen. Recently, it was verified that CXL maintenance monotherapy has a tendency to enhance a progression-free survival [2]. Currently, CXL was officially approved by State Food and Drug Administration of China (CFDA) for use as an oral capsule dosage to treat non-small-cell lung carcinoma(NSCLC) and breast cancers (Approval number: H20030359). The main anticancer mechanism of CXL is to damage the DNA templates of cancer cells, which leads to a considerable amount of DNA breaks and then results in cell death [1–2].

However, in preclinical, the efficacy of CXL decreased slowly after long term exposure in rats. There are many reasons that might be related to this phenomenon, such as the induction of Cytochromes P450 (CYP450) and the changes of drug “targets”, and so on. Currently, the mechanism is still not known. In this article, as one of the most important reasons, the possible induction of CYP450was investigated and the types of CYP 450 isoforms about induction were determined.

CYP450 are essential for the metabolism of many medications. According to statistics, CYP450 isoforms are responsible for the oxidative metabolism of approximately 85% of the marketed drugs [3]. Thus the induction and inhibition of CYP450 are one of the major concerns in preclinical and clinical practice. Induction of the drug metabolism is a process whereby the activity of enzymes responsible for drug metabolism is increased relative to their basal states within an individual. Unlike CYP450 inhibition, which is an almost immediate response, CYP450 induction is a slow regulatory process. It takes time to reach a higher steady-state enzyme level as a result of a new balance between the rate of biosynthesis and degradation. There are two major issues associated with CYP450 induction. First, induction may cause a reduction in therapeutic efficacy of comedications. For drugs whose effect is produced primarily by the parent drug, induction would increase the drug’s elimination, resulting in lower drug concentrations, and decrease the drug’s pharmacological effect. Second, induction may create an undesirable imbalance between detoxification and activation as a result of increased formation of reactive metabolites, leading to an increase in the risk of metabolite-associated toxicity and/or potential drug-drug interactions (DDIs) [4–5].

It is the purpose of this paper to investigate the possible auto-induction mechanisms. Three *in vitro* and *in vivo* rat models were used: a) the *in vitro* rat hepatocytes induction model; b) the *in vivo* ‘cocktail’ administration model and c) the pharmacokinetic study of the single and multiple doses.

## Materials and Methods

### Ethics statement

This study did not involve non-human primates. The experiments described in this article were performed in full accordance with the guidelines for animal experimentation released by the National Institute of Animal Health. This study was approved by the Animal Ethic Committee at Beijing Institute of Pharmacology and Toxicology (ETHICS CODE Permit NO. SCXK-(Beijing) 2007–004). Moreover, the approval was received prior to beginning the research.

### Materials

CXL was produced by Maoxiang Pharmaceutical Co. Ltd (purity > 99.3%, Tonghua, Jilin, China); Phenacetin, paraxanthine, dexamethasone (DEX), β-napthoflavone (BNF), 4-acetamiclophenol, 1-hydroxymidazolam and NADPH were obtained from Sigma-Aldrich Inc. (St. Louis, MO, USA); Caffeine, midazolam hydrochloride, tramadol hydrochloride and propranolol hydrochloride were bought from the National Institutes for Food and Drug Control (Beijing, China). Kolliphor HS15 was used as a solubilizer, granted from BASF China Company Ltd. (Beijing, China). Rat recombinant cytochrome P450 enzymes (CYP1A2 and CYP3A1) with reductase were purchased from BD Gentest® Corporation (Woburn, MA, USA). HPLC grade acetonitrile and methanol were obtained from Sigma–Aldrich Inc. (St. Louis, MO, USA). All other reagents and solvents were commercial products of analytical grade. All primers were synthesized by AuGCT Biotechnology (Beijing, China).

### Animals

The male Sprague-Dawley rats weight of 200 ± 20 g were obtained from the Vital River Laboratories (Beijing, China). The grades of these rats are Grade II. All of the animals were maintained under a 12 h light/dark cycle in standard cages and bedding with free access to standard commercial food and water for at least 1 week.

### *In vitro* evaluation of CYP450 induction using rat hepatocytes induction model

This procedure was performed as described previously [6–7]. Briefly, primary hepatocytes were isolated from adult Sprague-Dawley rats (200 to 220 g) by a modification of the two-step collagenase perfusion method. Then, the viability of rat hepatocytes was evaluated by trypan blue test and cell suspensions with a viability of >70% were applied. The viable hepatocytes were seeded onto matrigel coated 24-well plates, each well containing a cell density of 1 × 10^6^ viable cells in 1 mL of incubation medium. The incubation medium was Hepatozyme-SFM (Invitrogen, Carlsbad, CA) and was changed every 24 h throughout the entire experiment. Hepatocytes were first kept in a humidified CO_2_ incubator (37°C, 5% CO_2_) for 3 d before treatment with CXL. On d 4, the medium was aspirated and discarded, and then treated with different CXL (containing 0.1% DMSO) or the positive (PC, containing BNF or DEX) [8] or negative control (NC, containing 0.1% DMSO) for 3 d. At d 4, the total RNA was immediately extracted from the cells using TRIzol A^+^ reagent (Tiangen, China) according to the protocol of manufacturer. The real time polymerase chain reaction (RT-PCR) technique was conducted using an IQ5 RT-PCR detection system (Bio-Rad, Foster City, CA, USA) with TaqMan One-Step RT-PCR Master Mix Reagents containing 300 nM forward primer, 900 nM reverse primer, 200 nM TaqMan probe, and 25 ng of total RNA. The RT-PCR profile was as follows: 48°C for 25 min for reverse transcription, 95°C for 15 min for enzyme activation, 44 cycles of denaturation at 95°C for 15 sec, and annealing/extension at 60°C for 1 min [9]. The primers used in PCR amplifications were as follows [10]: CYP3A1 forward: TTCACCGTGATCCACAGCA and CYP3A1 reverse: TGCTGCCCTTGTTCTCCTT; CYP1A2 forward: CCTCACTGAATGGCTTCCACA, CYP1A2 reverse: TCTCATCATGGTTGACCTGCC; GAPDH forward: GTGGTGCCAAAAGGGTCAT and GAPDH reverse: ATTTCTCGTGGTTCACACCCA.

### The metabolism of CXL with recombinant CYP enzymes model

CXL dissolved in acetonitrile and diluted in the incubation buffer with the solvent concentration of 0.1%. 4 μmol/L of CXL was incubated separately in sodium phosphate buffer (100 mmol/L, pH 7.4) containing recombinant CYP1A2, and CYP3A1 coexpressed with cytochrome b5 in a final volume of 1 mL. Supersomes were used at P450 protein concentrations of 20 nmol/L. After preincubation for 5 min at 37°C, the reaction was initiated by the addition of NADPH (1.0 mmol/L). After 0, 10, 30 and 60 min, 100 μL was withdrawn from the incubation and added to 100 μL cold acetonitrile with tramadol (I.S., 100 ng/mL). The samples were mixed and placed on wet ice. After centrifuged with 14,000 g for 10 min, the supernatant was taken for LC-MS/MS analysis as described above. The incubations were performed in triplicate.

### *In vivo* ‘cocktail’ of CYP450 probe substrates and their metabolites model

Nine male rats were randomly assigned to a negative control group (NC), a positive control groups (PC) and an experimental group (n = 3). The experimental group was administered 60 mg/kg of CXL (containing 0.5% methylcellulose, *po*, twice daily), whereas the NC group was administered 0.5% methylcellulose. The PC group was administrated 25 mg/kg DXM (containing 0.5% methylcellulose, *po*, twice daily). At d 6, all of three groups were administered intraperitoneally the ‘cocktail’ probe substrates, which included caffeine (1 mg/kg) and midazolam (0.2 mg/kg). Blood samples (about 200 μL) were taken at 10, 20, 30 min and 1, 3 h. These blood samples were centrifuged at 3,500 × g for 10 min and then 50 μL of plasma sample was collected and then combined with 150 μL of acetonitrile (containing 20 ng/mL propranolol as the I.S.) as an internal standard (I.S.) and protein precipitation agent. The mixture was shaken vigorously for 1 min, centrifuged at 14,000 × g for 10 min (4°C), and 20 μL of each sample was injected into a HPLC-MS/MS system for the substrates and their metabolites analysis. The relative references were shown as described previously [11–13].

### HPLC-MS/MS for analysis of *in vivo* probe substrates and their metabolites

In this experiment, caffeine, paraxanthine (metabolite of caffeine), midazolam, 1-hydroxymidazolam (metabolite of midazolam) and propranolol (I.S.) were determined simultaneously by HPLC-MS/MS. The analysis method was similar with that of *in vitro* metabolites of the probe substrates. The positive ion multiple-reaction-monitoring (MRM) mode analysis was performed with the transitions m/z 195 → 138 for caffeine (the collision energy: 29 eV), the transitions m/z 181 → 124 for paraxanthine (the collision energy: 26 eV), the transitions m/z 326 → 249 for midazolam (the collision energy: 51 eV), the transitions m/z 342 → 324 for 1-hydroxymidazolam (the collision energy: 23 eV) and the transitions m/z 260→118 for propranolol (the collision energy: 23 eV).

### *In vivo* pharmacokinetics of single and multiple doses

In this experiment, the male rats (n = 6) were randomly assigned to single and multiple dose group and pharmacokinetics of CXL was investigated with oral doses of 60 mg/kg according to the human clinical regimen (20–30 mg/kg/d, tid). This dosing formulation (10 mg/mL) was prepared in 20% Kolliphor HS15 and administered orally at 6 mL/kg by gavage. For the single dose group, 200 μL blood samples were collected at 5, 15, 30, 45 min and 1, 1.5, 2, 4, 6, 8 and 12 h after the single administration. For the multiple dose group, the rats were administered the same dosage of CXL twice daily for the following 5 d. In order to achieve the steady state blood concentration, 200 μL trough concentration (C_min_) samples were also collected before each dosing. Finally, at the end of the multiple doses, blood samples were collected again at the same time points as the single dose regimen. These blood samples were centrifuged at 3,500 × g for 10 min and then 50 μL of plasma samples were collected and added 50 μL of tramadol solution (1 μg/mL in methanol) as the internal standard (I.S.). After adding 100 μL of protein precipitation agent (acetonitrile), the mixture was vortexed for 30 s then centrifuged at 14,000 × g for 10 min. The supernatant was transferred to clean vials for CXL analysis.

### Quantification of CXL in biosystems by LC/MSD

To quantify CXL, a LC/MSD quadruple mass spectrometer system (Agilent Co., Palo Alto, CA,USA) was used, which equipped with a binary pump, an automatic solvent degasser, an autosampler and a BetaBasic (Thermo Fisher Inc., Waltham, MA, USA) C18 column (2.1 mm i.d. × 150 mm, 5 μm). The mobile phase consisted of methanol—water—formic acid (65:35:0.1, v/v/v) with a flow rate of 0.2 mL/min. Detection was performed on an electrospray ionization (ESI) source operated under selected ion monitoring (SIM) mode. [M + H]^+^ at m/z 180 for CXL, and [M + H]^+^ at m/z 264 for tramadol (I.S.) were selected as detecting ions. The optimum ESI conditions included a nitrogen nebulizer with the pressure of 40 psi, nitrogen drying gas with the temperature of 300°C at 9 L/min, spray voltage of 4,500 V, a detector gain of 1600 V, and a fragmentation voltage of 100 V. The volume of each sample injected into the column was 10 μL. The retention times of CXL and tramadol were 2.7 and 1.9 min, respectively. CXL were quantified using the peak area ratios between the analyte and I.S. against the concentrations with weighted (1/x) least squares linear regression. The method was linear over a concentration range of 0.01 to 10.0 μg/mL.

### Data calculation and analysis

For *in vitro* evaluation of CYP450 induction, the relative (ΔCt) values of all samples were normalized to the glyceraldehyde 3-phosphate dehydrogenase (GAPDH) Ct value, and then the relative Ct values (ΔΔCt) of each sample, including CYP3A4 and CYP1A2 were obtained by comparison (ΔCt) of NC and PC. The relative gene expression rate was calculated using the (ΔΔCt) increase/decrease compared with the NC. Then, in order to determine EC_50_ and E_max_ values, concentration response data were fit to a three-parameter sigmoid (Hill) function with SigmaPlot 11.0 (Systat Software, Inc., Chicago, IL) and were calculated using the following equation [14]:

$$y=\frac{E_{\text{max}}\cdot X^{\gamma }}{EC_{50}^{\gamma }+X^{\gamma }}$$

For the *in vivo* induction assay, the phenotypic activities of CYP3A and CYP1A were determined with the use of 1-hydroxymidazolam/midazolam plasma ratios and paraxanthine/caffeine plasma ratios, respectively. Pharmacokinetic parameters in rat plasma were calculated using noncompartmental analysis by Gastroplus™ 8.5 software (n = 3).

All results were expressed as arithmetic mean ± standard deviation (SD). The Student’s *t* test was employed to compare the pharmacokinetic parameters of the single dose and multiple doses. The ANOVA analysis was used to evaluate statistically the mean differences of the phenotypic activities between the drug and NC or PC groups *in vitro* and *in vivo*. The Scheffe’s F post hoc test was applied as needed based on the results of the ANOVA analysis to identify individual differences.

## Results

### The induction on the *in vitro* rat primary hepatocytes studies

**Fig 2:**
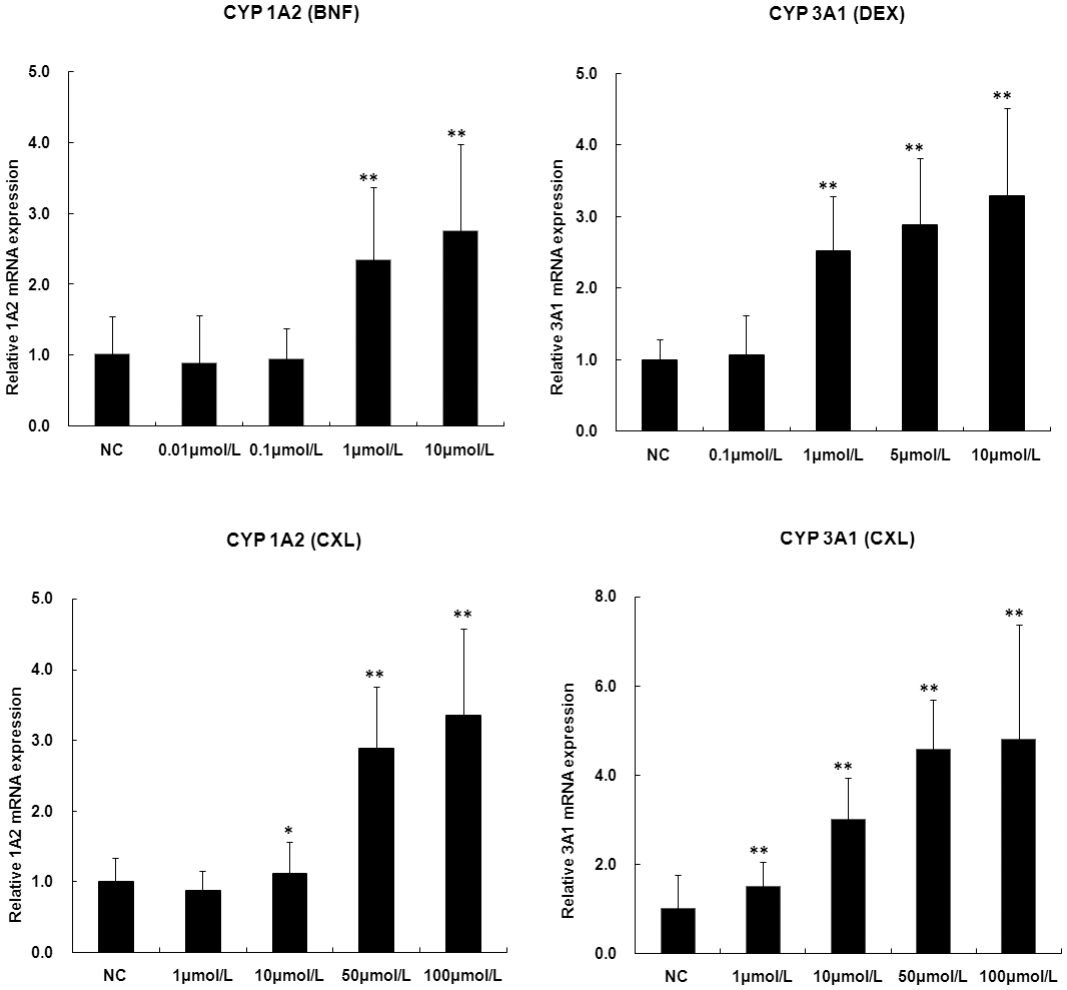
The *in vitro* induction study with fold of changes in the mRNA expression of the rat primary hepatocytes for CYP 1A2 and 3A1 isoforms from different treatment groups. (n = 3) (a) dexamethasone (DEX, 0.01–10 μmol/L); (b) β-napthoflavone (BNF, 0.1–10 μmol/L); (c)—(d) chloroxoquinoline (CXL, 1–100 μmol/L). (n = 3); *p<0.05, **p<0.01 vs. NC.

According to the results of the single and multiple doses, C_max_ of CXL in rats was about 64.8 ± 15.2 μmol/L after single-dose intragastric administration 60 mg/kg. Therefore, the concentrations of 1, 10, 50 and 100 μmol/L were chosen for the *in vitro* study. Meanwhile, before the induction study, the viability of rat hepatocytes was directly measured using the MTT cytotoxicity assay. CXL, when incubated with rat hepatocytes over the concentration range of 1–200 μmol/L, showed no reduction in cell viability indicating its non-toxic nature in this concentration range. According to the induction results, treated with CXL, NC and PC, the changes of CYP mRNA expression levels in the rat hepatocytes were shown in Fig 2. The expression of CYP3A1 (DEX) and CYP1A2 (BNF) was significantly increased in a concentration-dependent manner in all PC groups (Fig 2a-2b). The E_max_/EC_50_ values of induction were 13.5 and 14.6 for CYP1A2 and CYP3A1, respectively. As shown in Fig 2c, the expression levels of CYP1A2 in the middle-dose and high-dose (50 and 100 μmol/L) groups were significantly higher than that of low-dose (1 and 10 μmol/L) group. While the expression levels of CYP 3A1 were significantly increased in all groups (Fig 2d). The E_max_/EC_50_ values of CXL induction were 0.2 and 1.2 for CYP1A2 and CYP3A1, respectively. Therefore, it is implied that the biological induction of the CYP1A2 and CYP3A4 present in rats.

### Enzyme kinetics of CXL metabolism

The elimination half-life (t_1/2_) of CXL in recombinant CYP enzymes was calculated as t_1/2_ = 0.693/k, where k is the slope of the line obtained by linear regression of the natural logarithmic percentage (Ln %) of the remaining parent drug CXL versus the incubation time. The t_1/2_ of CXL incubated with rat recombinant CYP1A2 and 3A1 are 123.8 and 40.8 min respectively. The detail was shown in S1 Table.

### The CYP3A and CYP1A induction on the *in vivo* ‘cocktail’ studies

**Fig 3:**
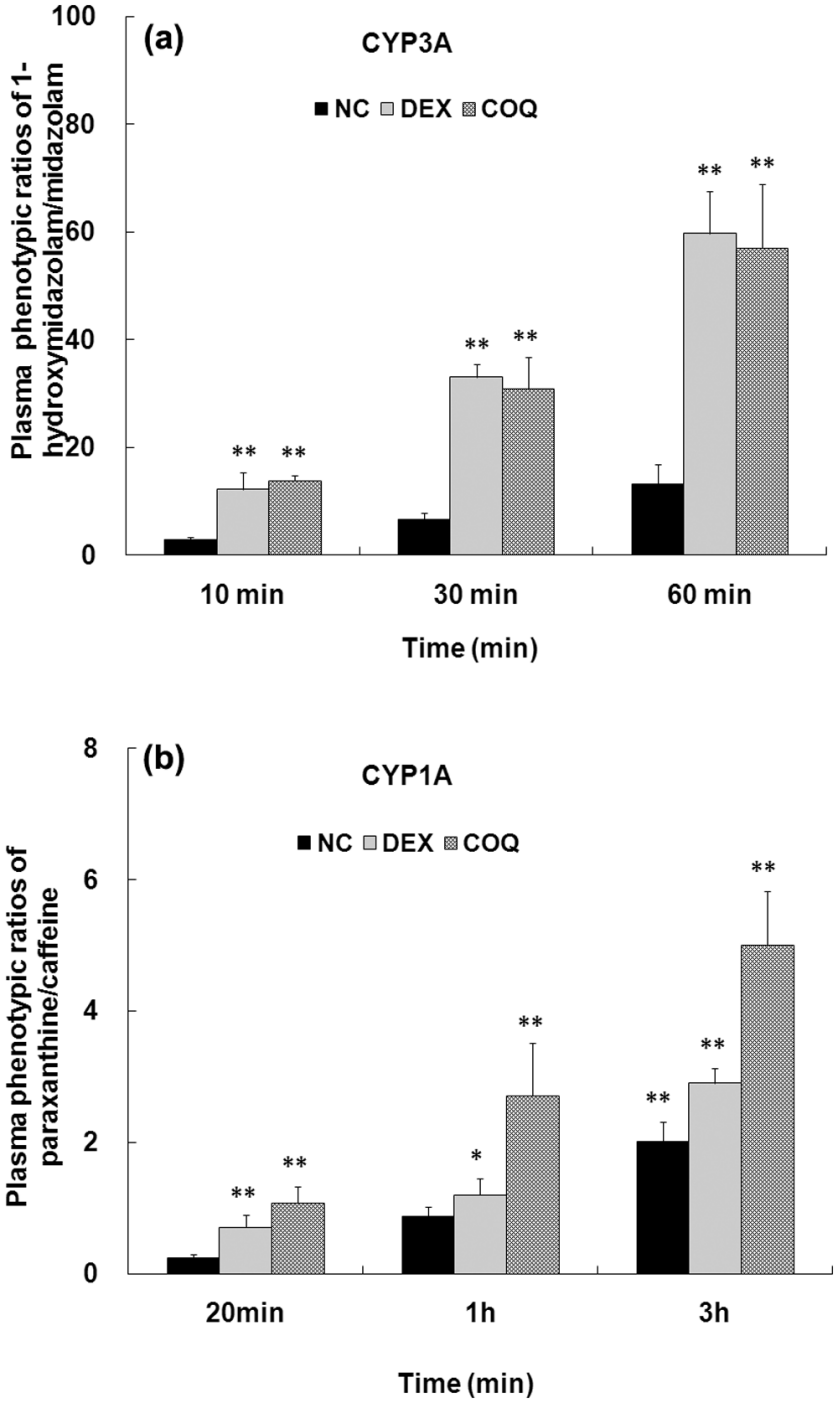
*In vivo* induction study of CYP3A and CYP1A with mixed phenotyping in rats: comparison of phenotypic ratios of 1-hydroxymidazolam/midazolam for CYP3A (a) and paraxanthine/caffeine for CYP1A (b). (i) 0.5% methylcellulose (NC); (ii) dexamethasone (DEX, 25 mg/kg, po, twice daily, PC); (iii) chloroxoquinoline (CXL, 60 mg/kg, po, twice daily). (n = 3); *p<0.05, **p<0.01vs. NC.

The effects of repeated pre-treatment with CXL (60 mg/kg) on CYP phenotypic ratios are shown in Fig 3. Compared to the NC, 5 d treatment with CXL resulted in a 4.4–4.8 times increase in the mean of 1-hydroxymidazolam/midazolam plasma ratios (*p*< 0.05, Fig 3a) at the time points of 10, 30 and 60 min. Similar to its effect on CYP3A, CXL produced the dramatic increase in CYP1A activity (*p*< 0.05, Fig 3b) after 5 d pre-treatment, which was a 248–450% increase in the mean paraxanthine/caffeine plasma ratios between CXL and NC at the time points of 20 min, 1 and 3 h. Meanwhile, multiple administration of DXM (PC) also produced significant changes in CYP3A and CYP1A phenotype. Therefore, compared to the NC and PC, CXL was implied to be a CYP3A and CYP1A inducer.

### The PK difference of the single and multiple doses

**Fig 4:**
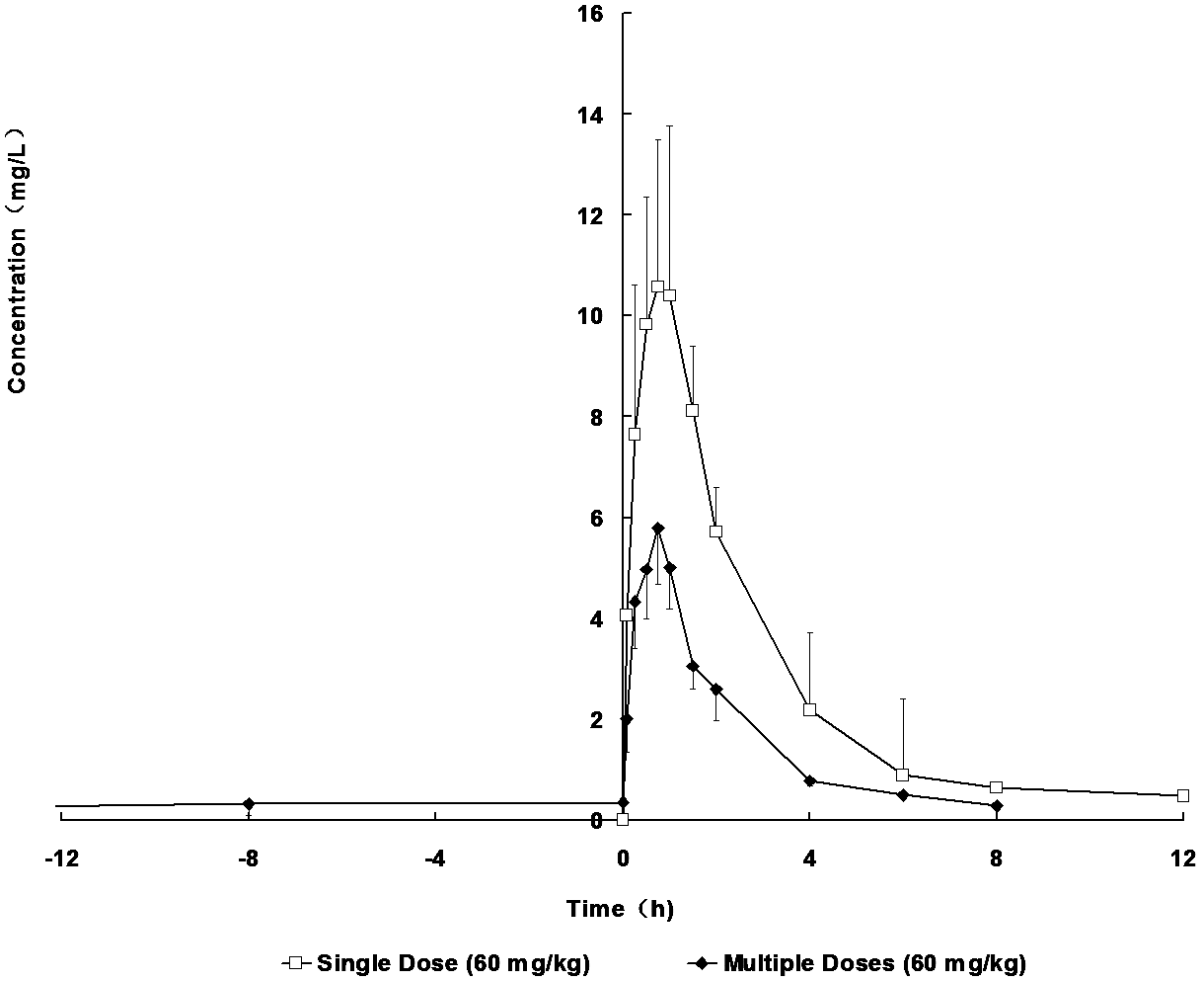
Concentration-time plots of CXL after single-dose and multiple-doses (*po*, 60 mg/kg) of CXL in rats (n = 3).

The concentration-time curves and the PK parameters after single and multiple doses of CXL in rats were shown in Fig 4, Table 1 and S2 Table. Compared with the single dose, AUC_0-t_ and C_max_ of the multiple doses were approximately 2.4-fold and 1.9-fold lower than those of the single dose, respectively. Meanwhile, there were statistically significant differences for AUC, C_max_, MRT and CL between the single and multiple doses of CXL. While, there were no statistically significant differences for V, T_1/2_ and T_max_. But, for multiple doses, T_1/2_ and V of multiple doses were higher than those of the single dose.

**Table 1:** PK parameters in rat plasma after single dose and multiple doses (intragastric administration, 60 mg/kg) of CXL (n = 3).

Parameters	Units	Single dose	Multiple doses	*p*
**AUC**_**(0–12)**_ **	mg/L*h	31.3 ± 2.4	13.3 ± 1.9	0.0005
**AUC**_**(0-∞)**_ **	mg/L*h	37.4 ± 4.2	14.2 ± 2.0	0.00101
**MRT**_**(0–12)**_ *	h	2.6 ± 0.2	2.0 ± 0.0	0.01015
**t**_**1/2**_	h	8.0 ± 4.8	2.4 ± 0.3	0.10942
**CL** **	L/h/kg	1.6 ± 0.2	4.3 ± 0.6	0.00222
**V**	L/kg	18.0 ± 8.9	14.6 ± 0.9	0.54352
**Cmax** *	mg/L	11.9 ± 2.5	6.1 ± 0.6	0.01748
**Tmax**	min	0.8 ± 0.3	0.7 ± 0.1	0.64333

* p<0.05,

** p<0.01 vs. NC.

## Discussions

In general, the CYP450 induction is less likely to result in safety issues and it may decrease efficacy of one or more medications. Therefore, drug-drug interactions mediated by CYP450 induction are paid less attention than those mediated by P450 inhibition. But several studies indicated that the induction could result in liver toxicity according to the histopathology of hepatocytes in animal toxicology research [15]. Therefore, induction has caused more and more concerns, especially in CYP 450 induction. In this paper, the induction potential of CXL was assessed using three rat models. The results were of great benefit to clarify the possible induction mechanisms.

Meanwhile, the induction potential of new molecular entities (NMEs) is easy to assess *in vitro*, however it is not necessarily predictive *in vivo* due to the complexity *in vivo*. Therefore, three *in vitro* and *in vivo* models complemented each other in this research. In study 1, mRNA expression levels of CYP3A1 and CYP1A2on the *in vitro* rat primary hepatocytes model was first determined by using the RT-PCR technique. Consequently, the expression of CYP3A1 and CYP1A2 was significantly increased in a concentration-dependent manner in middle-dose and high-dose (10, 50 and 100 μmol/L) groups. In study 2, the mixed phenotyping (‘cocktail’) model *in vivo* was used. The results showed that CXL could produce significant induction of CYP3A and CYP1A phenotype according to the results of up-regulated paraxanthine/caffeine and 1-hydroxymidazolam/midazolam plasma ratios after multiple administrations. To further verify the underlying mechanisms of induction, in study 3, repeated administration of CXL at doses up to 60 mg/kg in rats had significantly changed pharmacokinetics parameters, such as decreased AUC and C_max_ from single to the multiple dosing regimens.

Additionally, the selective substrate probes of CYP isoforms are valuable tools allowing measurement of the *in vivo* CYP activity of preclinically, clinically and toxicologically important enzymes [16]. There are generally two *in vivo* methods for identifying the enzyme phenotype. One method involves using one single probe drug, whereas the other is a mixed phenotyping (‘cocktail’) method with simultaneous administration of multiple types of CYP-specific probes [11]. Compared to the single probe method, the approach of the ‘cocktail’ administration can simultaneously provide independent phenotypic measures for multiple CYP enzymes, which is more suitable for the high throughput screening of NMEs [12]. However, the use of ‘cocktail’ approach requires that there is no inter-substrate interaction. Therefore, midazolam and caffeine were selected because the inter-substrate interaction was not observed between them [13]. It is also worth remembering that CYP1A2 of rat was found to be a key enzyme catalyzing 8-hydroxylation (72%) and substantially contributing to 3-N-demethylation (47%) and 1-N-demethylation (37.5%) of caffeine. Therefore, 1,3,7-trimethyluric acid (the metabolite of 8-hydroxylation) is better than paraxanthine (the metabolite 3-N-demethylation) to indicate the CYP 1A2 activity in rats [17]. But due to the difficult availability of 1,3,7-trimethyluric acid in the Chinese market, paraxanthine was still chosen in this experiment. Additionally, as an observation index of enzyme activity, metabolite/probe plasma ratio is better than the plasma concentration of metabolite, due to it reflecting the decrease of probe concentration and the increase of metabolite concentration at the same time.

Nevertheless, in this manuscript our observation only paid attention to the induction effect on CYP 1A and 3A, it was unclear if other CYP subtypes were also involved in CXL auto-induction. Moreover, these results from rat models cannot extrapolate simply to the human. Therefore, to establish a correlation between data generated *in vitro* and data observed in clinical studies, an *in vitro* human hepatocytes cell-based study is of great importance in the next step.

In conclusion, using *in vitro* and *in vivo* rat models simultaneously, our studies here provide the first evidence of an association between repeated administration of CXL and induction of CYP1A and 3A in rats. Although species difference may exist, it is implied that the biological induction of the CYP 1A and CYP 3A present in the range of the effective doses of CXL for rats, which is more likely to lead to the decreased efficacy after long term exposure. Therefore, attention should be paid in the further studies in preclinical and clinical.

## Supporting Information

S1 Table CXL metabolism in reactions with rat recombinant CYP3A4 and CYP 1A2 enzymes and the percentage of CXL remaining versus the incubation time (n = 3). (DOCX)

S2 Table Concentration-time data of CXL after single-dose and multiple-doses (*po*, 60 mg/kg) of CXL in rats (n = 3). (DOCX)
