# Supplementary material for: Auto-Induction Effect of Chloroxoquinoline on the Cytochrome P450 Enzymes of Rats Associated with CYP 3A and 1A
Source: PLoS One. 2015 Sep 23;10(9):e0138875. doi: 10.1371/journal.pone.0138875 (PMC4580629; doi:10.1371/journal.pone.0138875)
Supplement: S1 Table — (DOCX) [file pone.0138875.s001.docx]

S1 Table CXL metabolism in reactions with rat recombinant CYP3A4 and CYP 1A2 enzymes and the percentage of CXL remaining versus the incubation time (n = 3)

| Time (min) | Concentration (µg/mL) | |  | Remaining (%) | |
| --- | --- | --- | --- | --- | --- |
|  | CYP 3A4 | CYP 1A2 |  | CYP 3A4 | CYP 1A2 |
| 0 | 4.59 ± 0.17 | 4.50 ± 0.15 |  | 100.0 ± 3.8 | 100.0 ± 3.3 |
| 10 | 3.35 ± 0.63 | 4.15 ± 0.17 |  | 73.0 ± 13.7 | 92.2 ± 3.8 |
| 30 | 2.65 ± 0.58 | 3.76 ± 0.28 |  | 57.7 ± 12.6 | 83.5 ± 6.1 |
| 60 | 1.56 ± 0.22 | 3.19 ± 0.22 |  | 34.0 ± 4.7 | 71.0 ± 4.9 |
