# Supplementary material for: Auto-Induction Effect of Chloroxoquinoline on the Cytochrome P450 Enzymes of Rats Associated with CYP 3A and 1A
Source: PLoS One. 2015 Sep 23;10(9):e0138875. doi: 10.1371/journal.pone.0138875 (PMC4580629; doi:10.1371/journal.pone.0138875)
Supplement: S2 Table — (DOCX) [file pone.0138875.s002.docx]

S2 Table Concentration-time data of CXL after single-dose and multiple-doses (*po*, 60 mg/kg) of CXL in rats (n = 3)

| Time | Concentration (µg/mL) | |
| --- | --- | --- |
|  | Single Dose | Multiple Doses |
| -24 h | - | 0.18 ± 0.06 |
| -16 h | - | 0.23 ± 0.05 |
| -8 h | - | 0.31 ± 0.13 |
| 0 h | - | 0.34 ± 0.09 |
| 5 min | 4.06 ± 2.96 | 2.00 ± 0.66 |
| 15 min | 7.65 ± 2.53 | 4.33 ± 0.92 |
| 30 min | 9.83 ± 2.91 | 4.96 ± 0.96 |
| 45 min | 10.58 ± 3.36 | 5.78 ± 1.11 |
| 1 h | 10.40 ± 1.29 | 5.00 ± 0.82 |
| 1.5 h | 8.11 ± 0.89 | 3.05 ± 0.47 |
| 2 h | 5.72 ± 1.54 | 2.59 ± 0.62 |
| 4 h | 2.18 ± 1.50 | 0.78 ± 0.10 |
| 6 h | 0.89 ± 0.08 | 0.50 ± 0.03 |
| 8 h | 0.64 ± 0.08 | 0.28 ± 0.02 |
| 12 h | 0.48 ± 0.12 | - |
